# Predicting guide dog career success using machine learning and large language models

**DOI:** 10.1038/s41598-026-48238-3

**Published:** 2026-04-24

**Authors:** Adir Solomon, Elena Shamis, Hadas Racah, Amihai Toren, Carmel Weiss, Orna Braun, Anna Zamansky

**Affiliations:** 1 Information Systems Department, University of Haifa, Haifa, Israel; 2 Israel Guide Dogs Center for the Blind, Beit Oved, Israel

**Keywords:** Computational biology and bioinformatics, Mathematics and computing

## Abstract

Guide dogs play essential roles in supporting independence and well-being of visually impaired people. High attrition rates underscore the need for more effective early prediction of dog suitability for a guide dog career. This study presents a novel data-driven framework that integrates machine learning and large language models to predict guide dog training outcomes using data from the Israel Guide Dog Center (IGDC). The dataset included structured Behavior Checklist (BCL) assessments and unstructured trainer textual comments collected from 990 dogs at the pre-training stage. Incorporating trainer comments alongside structured behavioral assessments led to a substantial improvement in predictive performance compared to models based on BCL data alone. Furthermore, the pipeline generated interpretable textual explanations of model predictions, which were empirically evaluated by two experienced guide dog trainers. The findings highlight the potential of combining structured behavioral metrics with natural language data to enhance predictive accuracy and decision support in guide dog selection and training.

## Introduction

Working dogs support public safety, health, and daily living across roles that include guide work, assistance tasks, detection, and emergency response. Performance in these settings depends on stable behavior, strong trainability, and task-appropriate temperament, together with extended, intensive training^1–3^. Despite the strategic importance of working dogs, a significant proportion of candidates fail to meet the behavioral and performance standards necessary for deployment. Many of these unsuitable dogs are not identified until they are one to two years old, after substantial investments of time, financial resources, and training efforts have already been made. Training success in working and assistance dog programs is often limited by high attrition rates. Research indicates that a substantial proportion of candidates, frequently between 50% and 70%,do not meet the necessary standards for deployment. Behavioral or temperament-related issues are the most common reasons for release. The problem is particularly pronounced when dogs are recruited from shelters, where failure rates can approach 80%, underscoring the influence of early life environment and genetics on program suitability^4–7^.

Early identification of dogs with the greatest potential for success is not only a matter of cost-efficiency. It also has important animal welfare benefits. Dogs that are a poor fit for working roles may experience stress or frustration during training; identifying them early means they can be redirected to more appropriate homes or careers sooner, avoiding prolonged failure and allowing them to thrive elsewhere. Ultimately, enhancing dog screening and assessment strategies strengthens the reliability and ethical integrity of working dog programs, while expanding the availability of highly capable canine partners for critical societal roles.

Guide dogs provide individuals with visual impairments not only with reliable mobility assistance but also with greater independence in daily life. Beyond their practical function, guide dogs often contribute to improved social engagement and emotional well-being, serving as a complement to, rather than a replacement for, conventional mobility tools^8^.

Guide-dog organizations invest heavily in breeding, socialization, and training to meet high professional standards. These efforts require experienced staff, dedicated facilities, and long-term development from early puppyhood through advanced skills. The cost of preparing a single qualified guide dog has been estimated on the order of tens of thousands of U.S. dollars^9^. In addition, limited qualification rates further increase financial and logistical demands^10,11^. Because many well-cared-for dogs are ultimately rehomed or redirected to other roles, better early prediction remains essential for efficiency and cost-effectiveness.

With the aim of improving efficiency and reducing production costs, factors that could predict a dog’s suitability for guiding work have been extensively studied. The Canine Behavioral Assessment and Research Questionnaire (C-BARQ) is an owner/handler survey that records dogs’ behavior across everyday contexts and has been used to follow development in guide-dog programs^4,12,13^. C-BARQ has been shown to provide valuable insights into behavioral development and has been widely adopted in both research and practice. However, C-BARQ is typically completed by puppy raisers or owners prior to the onset of formal training and reflects behavior in non-institutional, home-based environments. In contrast, the present study focuses on decision-making at the point of entry into structured guide dog training, where assessments are conducted by professional trainers in a standardized training context. For this operational stage, trainer-based evaluations such as the Behavior Checklist (BCL), together with qualitative trainer observations, are more directly aligned with the goal of reducing the number of dogs that enter training but are later released, and the associated costs^14^.

The BCL is a structured tool used by guide-dog programs to evaluate a wide range of canine behavioral traits. It includes 52 items designed to capture reactions related to emotional regulation, stress, fear, social interactions, and responsiveness to environmental stimuli. These items are organized into thematic categories that correspond to common behavioral challenges encountered during training, such as adaptability, resilience, and touch sensitivity.

Each behavior is scored on a scale from one to five, with lower scores indicating a more pronounced expression of the trait. For instance, in assessing fear of unfamiliar people, a dog that confidently engages would receive a high score, while one that exhibits avoidance or distress would be rated lower. This approach enables trainers to monitor progress and identify potential concerns over time. In routine practice, the BCL is administered repeatedly at standardized stages of a dog’s development and training. This allows trainers to compare scores across assessment time points and monitor changes in behavioral traits over time, thereby identifying emerging concerns or improvements.

The BCL has since been widely adopted by guide-dog organizations worldwide and is rapidly emerging as the standard behavior assessment during training^15^. Its structured, standardized evaluation of behavioral traits supports improvements in guide-dog selection and training outcomes. The Israel Guide Dog Center (IGDC) is among the organizations that use the BCL. It is important to note that its validation is primarily based on extensive institutional application and professional consensus among international guide-dog organizations rather than traditional psychometric validation frameworks. This long-term operational history ensures the tool is directly aligned with the specific behavioral requirements and practical outcomes of guide-dog programs. In the present study, we focus on a single BCL assessment conducted at the point of entry into formal guide dog training, which serves as the basis for our predictive modeling.

In Israel, the number of legally blind and visually impaired individuals is estimated at approximately 24,000. The IGDC is able to support this community by providing trained guide dogs that promote independence, mobility, and quality of life. The center adheres to international standards and collaborates with global partners to maintain high levels of training and care. Over the decades, the center has developed a comprehensive database that informs internal breeding and training decisions. This database includes BCL scores and related behavioral and training information for nearly one thousand dogs, which provides the basis for the present study.

All of the above-mentioned scoring and assessment methods have one inherent limitation: the subjectivity inherent to human scoring. Integrating data-driven techniques for decision support on working dog suitability can complement and enhance traditional methods by reducing bias, increasing consistency, and supporting more objective and reliable assessments. It is thus not surprising that the use of machine learning is beginning to be explored in this domain.

Recent studies have explored data-driven approaches for predicting training outcomes in working dogs using a variety of data sources. One line of work focuses on smart collar technologies equipped with inertial measurement units (IMUs) to capture detailed movement and behavioral signals. Wu et al.^16^ demonstrated that accelerometry data from such sensors can be used to train deep learning models to distinguish gait patterns relevant to training assessments. Building on this approach, Martin et al.^17^ applied convolutional long short-term memory (Conv-LSTM) networks to classify specific behaviors from IMU-based sensor data, highlighting the potential of wearable technologies for objective behavioral monitoring. Mealin et al.^14^ similarly relied on IMU data, applying a neural network to predict BCL scores across behavioral categories such as noise sensitivity and body awareness, achieving high predictive accuracy.

A complementary line of research applies machine learning techniques to structured behavioral surveys and trainer-reported assessments rather than sensor data. Eyre et al.^18^ examined dogs enrolled in olfactory detection training programs and predicted whether they would succeed in pre-training or be dismissed due to behavioral issues using traditional machine learning models, including logistic regression, support vector machines (SVM), and random forests. More recently, Amirhosseini et al.^19^ predicted training outcomes in assistance dogs using behavioral survey data derived from C-BARQ assessments. After applying principal component analysis (PCA), they found that SVM and XGBoost outperformed deep learning models across two large datasets. Taking a broader perspective, Stevens et al.^20^ combined dog-specific behavioral traits with owner characteristics to predict success in the American Kennel Club Canine Good Citizen training program, identifying low disobedience as a strong predictor of success.

The rapid development of large language models (LLMs), including systems such as GPT, has opened new possibilities for data-driven decision support across disciplines. These models can process and generate natural language with a high degree of fluency, enabling their use in a variety of complex analytical and communicative tasks^21^. LLMs have already demonstrated potential in diverse fields, including finance^22^, education^23^, and healthcare^24,25^.

LLMs are increasingly being adapted for applications beyond traditional text processing, including use cases in animal behavior analysis and pet interaction. For instance, recent tools such as AmadeusGPT^26^ have enabled real-time, natural language interaction for behavior interpretation, allowing users to define and analyze pet behaviors through conversational prompts. Similarly, systems developed by Guo et al.^27^ have demonstrated how LLMs can simulate aspects of pet companionship, supporting user interaction through dialogue, scheduling, and even emotional engagement. These systems illustrate the versatility of LLMs, particularly in supporting mental and emotional well-being^28^. Building on these capabilities, Heiligers et al.^29^ developed a method for generating pet adoption descriptions using GPT-3.5. Xue et al.^30^ developed a system that uses LLMs to analyze visual and auditory data from smart collars in real time.

The main objective of this study is to explore novel ways for enhancing machine learning–based analyses of BCL scores through the integration of LLMs. Specifically, we aim to examine how textual information, in the form of qualitative trainer comments recorded at various stages of the training process, can be leveraged alongside quantitative BCL scores to improve predictive accuracy and interpretability. To this end, we utilize the IGDC database to first develop a baseline predictive machine learning model relying solely on structured BCL data. We then investigate the added value of incorporating unstructured text data, applying natural language processing techniques and LLM-based embeddings to capture contextual and behavioral nuances that may not be reflected in numerical scores alone. This combined approach can yield more accurate predictions of training success and provide deeper insights into the behavioral factors influencing guide professionals’ decision-making.

## Methods

All procedures were conducted in accordance with relevant guidelines and regulations. The study was based on historical data obtained from the Guide Dog Center and was granted exemption from ethical approval by the University of Haifa Ethical Committee.

### The IGDC database

At the Israel Guide Dog Center (IGDC), dogs demonstrating exceptional behavioral and physical traits are initially selected for the breeding program to ensure the continuity of high-quality working dog lines. Following the breeding assessment, if a dog is deemed unsuitable for guide dog work, it is evaluated for potential placement as a service dog in alternative assistance roles. Dogs that do not meet the criteria for either guide or service work are subsequently discharged from the program.

The IGDC database contains comprehensive historical records covering each dog’s health, behavioral assessments, and training progression from birth through the end of its life. Among these records are repeated BCL evaluations, conducted by center trainers at multiple key stages of a dog’s development. In the present study, we focused specifically on the main BCL evaluation prior to the commencement of formal guide dog training, referred to as the “*in for training”* assessment. This evaluation is typically conducted when the dog returns from its foster family and is about to enter the structured training program. It serves as a critical checkpoint, providing a consolidated profile of the dog’s behavioral readiness, temperament, and potential suitability for guide work. Because it is the last behavioral assessment before intensive training begins, this time point is particularly valuable for predictive modeling, offering a snapshot of traits most relevant to determining future training success or release from the program. It is also used for making breeding decisions.

In addition to the structured BCL scores, IGDC trainers also usually record free-text comments following this assessment, capturing nuanced observations about the dog’s behavior, responses, and environmental context. These narrative insights often reflect subtleties not fully encapsulated by the numerical scores, such as specific situational reactions or emerging behavioral patterns. Analyzing these comments will be central to our exploration.

### The dataset

In our dataset, each dog was assigned one of four final outcomes: *Guide Dog*, *Breeding*, *Service Dog*, or *Unsuitable*. For modeling purposes, we defined a binary target variable, where dogs classified as *Guide Dog* or *Breeding* were labeled as **successful** (label = 1), and those labeled as *Service Dog* or *Unsuitable* were considered **unsuccessful** (label = 0). This labeling scheme resulted in 314 successful and 676 unsuccessful instances.

Each dog was described by:

1. In-for-training BCL scores, overall 52 categories (the standard BCL form can be found here). In our given dataset, only 48 of these items were consistently recorded and available for analysis, while the remaining items contained systematic missing values and were therefore excluded.
2. Demographical information on breed, sex and lineage.
3. Textual comments columns.

Missing values in numerical fields were computed using the median, while missing categorical values were replaced with the placeholder “unknown.”

**Table 1:** Categorical features with demographic, hereditary, and qualitative assessments.

**Feature**	**Description**
Breed	Golden Retriever/Labrador/Mix
An innate desire to work	Trainer-assessed motivation to perform tasks
Walking speed	Typical walking pace during training sessions
Transition from walk to trot	Smoothness of gait transition
Sex	male/female
Father	dog identifier
Mother	dog identifier

**Table 2:** Free-text fields in the dataset based on trainers’ comments.

**Text Field**	**Description**
Summary of assessment procedures	Narrative summary of formal evaluation steps
BCL MA	Supplementary notes on the Behavioral Checklist test
Dog health status	Observations on physical or medical condition
Staying Alone Test	Behavior observed during short-term isolation
Additional considerations	Extra notes regarding temperament, special needs, or incidents
General comments on the evaluation	Overall assessment, often including subjective impressions

### The high-level approach

**Fig. 1:**
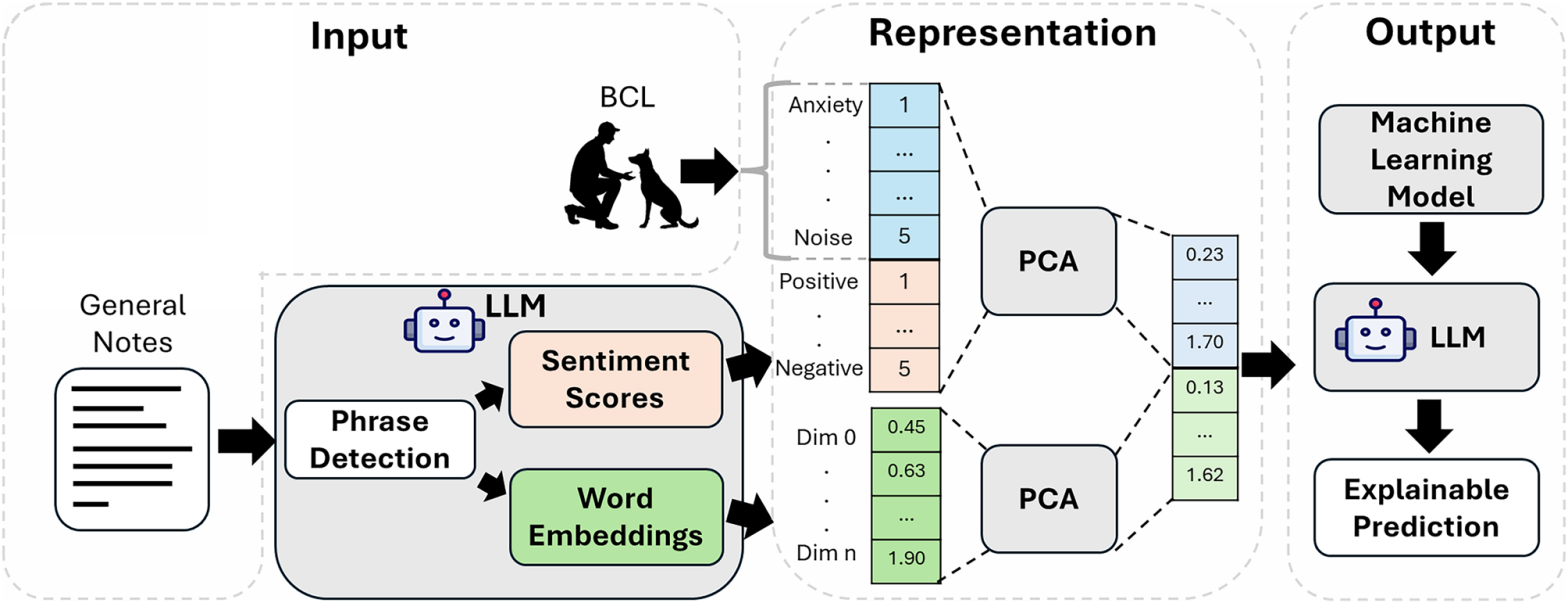
Overview of our method.

Figure 1 provides a high-level view of the proposed pipeline, which integrates structured behavioral assessment data (BCL scores) with unstructured textual observations (trainer notes), the latter transformed into numerical representations using a large language model (LLM). Our hypothesis was that the free-text comments may capture nuanced behavioral insights that are not fully reflected in the structured BCL features. To test this, first develop a baseline machine learning model that relies solely on the structured information extracted from the BCL scores of each dog (shown on the upper part of the figure). This baseline serves as a reference point for subsequent performance gains. In the next stage, we incorporate an additional information layer derived from free-text trainer notes (shown on the lower part of the figure). These textual descriptions are processed via an LLM to generate numerical embeddings that capture semantic and contextual information, which is then provided as input features to the supervised classification model. Finally, the structured and unstructured feature sets are fused into a unified representation and fed into the classification model, which outputs a binary prediction indicating whether the dog is deemed suitable for the intended working role.

### The baseline model

We use CatBoost^31^, a gradient boosting framework based on decision trees, known for its high performance on tabular datasets and its ability to handle categorical variables natively without the need for explicit encoding. This makes it particularly well-suited to our dataset, which includes both numerical and categorical features.

### Integrating textual information

In our framework, the LLM is used solely for transforming unstructured trainer comments into numerical feature representations. All suitability predictions are generated by the supervised machine learning model described below; namely, the LLM does not perform classification or decision-making.

**Sentiment Scores.** The feature engineering process involved four main stages. First, we use an LLM to decompose each text entry into atomic semantic units, short phrases representing single behavioral observations. For instance, the sentence “The dog sometimes fears stairs but gets along well with people” was split into two distinct phrases: “sometimes fears stairs” and “gets along well with people.” Second, each atomic phrase was classified by the LLM as having a positive, negative, or neutral sentiment, based on its implications for success in guide dog training. Third, the LLM identified modifiers in each phrase that indicated the intensity of the described trait (e.g., “slightly,” “very,” “extremely”). Finally, these modifiers were normalized to a 7-point ordinal scale, capturing the strength of the sentiment from “very weakly” to “extremely.”

Based on the structured output from the LLM, we computed several aggregated indicators for each dog: the number of positively framed phrases, the number of negatively framed phrases, the summed intensity of positive expressions, the summed intensity of negative expressions, and a final cumulative sentiment score, capturing the overall tone of the textual assessment. We concatenated these features with the original 48 numerical features. The combined feature space was then compressed to 15 principal components using PCA, preserving 87.1% of the total variance.

All stages of this process were performed automatically by the LLM using predefined prompts, without manual annotation or post-processing.

**Word Embeddings.** To further capture latent semantic information, we constructed a final model incorporating dense sentence embeddings. Each atomic phrase extracted from the text was encoded into a high-dimensional vector using the SentenceTransformer model (https://huggingface.co/sentence-transformers/multi-qa-mpnet-base-dot-v1.). For each dog, the phrase embeddings were averaged to produce a single semantic representation vector. To manage dimensionality and ensure balance between feature sources, we applied separate PCA transformations to the different feature sets. The embedding vectors were reduced to five principal components, while the numerical and LLM-derived features were reduced to ten components. These two sets were then concatenated to form a final 15-dimensional representation for each dog.

### Generating explanations

To generate explanations for the classification of the dogs based on the BCL and the general notes, we used an LLM in conjunction with the probability output of the machine learning model to produce interpretable justifications for its predictions. The prompt employed in our experiments was as follows:

“You are an expert in dog behavior and guide dog training. This is about a dog named {name} who is {suit} for the role of a guide dog. The dog’s behavior was evaluated using a predictive model, which estimated that the dog would be able to work as a guide dog with a probability of {Machine Learning Model Probability}. As an expert, explain the model’s prediction using the trainer’s notes on the dog’s behavior. Focus on the main issues related to guide dog suitability: {Issues}. Briefly and generally mention other relevant issues, if any. Use only the handler’s notes on the dog, do not add information that is not included in the notes. Be concise in your response. Your explanation will be read by experienced handlers; therefore, provide only a general description of the dog’s qualities without unnecessary detail.”

We present here an example of one of the explanations generated by the LLM for the dog Julio 1696:

Based on the model’s prediction, the probability that Julio 1696 is suitable to serve as a guide dog is higher than average (0.68). According to Julio 1696’s BCL data, it can be observed that:

- Overactivity due to stress, separation anxiety, inability to focus or concentrate, noise sensitivity, and distraction by other dogs received the highest score of 5.0.
- Anxiety in unfamiliar places, poor self-regulation, and lack of initiative received scores of 4.0.
- Avoidance and inhibition due to stress and physical sensitivity to touch with objects received scores of 3.0.

According to the trainer’s notes, Julio 1696 shows poor self-regulation under stressful situations, with resistance to collar and harness pressure, including freezing and refusal to move at first. There is physical sensitivity to contact with equipment and mild stress responses, such as lowering of the hindquarters and braking behavior. Mild avoidance and moderate sensitivity toward new stimuli (e.g., statues, unfamiliar dogs) were observed, with a need for reassurance, as well as slight excitement in social contexts and when playing with toys. He shows good food drive but is somewhat distant from physical contact. No signs of separation anxiety, overactivity due to stress, inability to focus, noise sensitivity, anxiety in unfamiliar places, distraction by other dogs, or lack of initiative were observed. No additional general issues were noted.

## Experimental settings

**Evaluation Metrics.** To comprehensively evaluate model performance, we employed the following metrics, each capturing a different aspect of predictive quality:

- **ROC-AUC (Area Under the Receiver Operating Characteristic Curve)**: This metric quantifies the model’s ability to distinguish between successful and unsuccessful dogs by measuring the area under the curve formed by plotting the true positive rate against the false positive rate across all possible classification thresholds.
- **PR AUC (Area Under the Precision-Recall Curve)**: This metric summarizes the trade-off between precision and recall across various classification thresholds. It is especially appropriate for imbalanced classification tasks, as it focuses on the model’s ability to correctly identify the minority class (in this case, successful guide dogs), rather than being influenced by the majority class.
- **F1-score**: The harmonic mean of precision and recall, this metric provides a balanced measure that accounts for both false positives and false negatives.
- **Accuracy**: The proportion of all instances that are correctly classified by the model. It is computed as the number of correct predictions (both successful and unsuccessful dogs) divided by the total number of evaluated cases.
- **Precision**: This metric reflects the proportion of correct positive predictions among all instances classified as positive. High precision indicates a low false positive rate.
- **Recall**: Also known as sensitivity or true positive rate, recall measures the proportion of actual positive cases that were correctly identified by the model.

In addition to these standard predictive metrics, we also assessed *economic efficiency* to translate abstract performance measures into business-relevant indicators:

- **Screened**: The percentage of screened-out unsuitable dogs. This is a key cost-saving measure indicating the proportion of dogs predicted as unsuitable that would indeed fail final certification (target = 0), thereby avoiding unnecessary and expensive training.
- **Training Pass**: The percentage of dogs selected by the model for training, reflecting the overall reduction in training admissions due to the screening process.

This combined evaluation framework enables a comprehensive comparison of models, considering not only predictive accuracy but also the operational and economic implications of their deployment in the guide dog selection process.

**Evaluation of Explanations.** To evaluate the quality of the explanations generated by our method, we collaborated with two experienced dog trainers, each with over 20 years of professional experience. Both trainers were familiar with the 11 dogs included in the classification results and were therefore well-positioned to assess the explanations and determine how realistic they were.

The evaluation procedure followed approaches similar to prior studies^32–35^ that examined explanations generated by LLMs. Each trainer was asked to complete a questionnaire consisting of several evaluation criteria, with each criterion rated on a 5-point Likert scale. The evaluated dimensions and their corresponding questions were as follows:

- **Comprehensiveness** – Assesses whether the explanation covers all key elements related to the dog’s suitability (e.g., important BCL features, notable trainer notes).
- **Clarity** – Measures how clearly the explanation is written, using appropriate professional terminology.
- **Relevance** – Evaluates whether the explanation aligns with the actual data about the dog.
- **Usefulness** – Examines the practical value of the explanation in assisting trainers’ decision-making.

The detailed measurement criteria, questions, and descriptions provided to the trainers are presented in Table 3.

**Table 3:** Evaluation criteria for generated explanations.

**Criterion**	**Question**	**Description and Scale (1–5)**
Comprehensiveness	Does the explanation address all the main components relevant to the dog’s suitability decision? (e.g., key BCL characteristics, notable trainer comments)	1 = Not comprehensive at all – misses most important factors, ignores critical reasons. 2 = Not comprehensive – covers only a small portion of relevant reasons. 3 = Moderately comprehensive – includes some important factors but omits major ones. 4 = Almost comprehensive – covers most main reasons, with only minor omissions. 5 = Fully comprehensive – addresses all main factors in sufficient detail.
Clarity	Is the explanation written in simple, clear language understandable to trainers, using accepted professional terminology?	1 = Not clear at all – difficult to follow, overly technical or vague. 2 = Unclear – understandable only with effort; partially confusing language. 3 = Neutral – somewhat clear but includes vague or inconsistent terms. 4 = Clear – generally easy to understand, with only minor issues. 5 = Very clear – crystal-clear, concise, and fully understandable.
Relevance	Does the explanation contain information inconsistent with the actual data about the dog?	1 = Not relevant at all – mostly unrelated, generic, or inconsistent with records. 2 = Not relevant – large portions are unrelated; minimal connection to data. 3 = Partially relevant – mix of specific and general content; not always accurate. 4 = Mostly relevant – focused on the case with minor unrelated content. 5 = Fully relevant – entirely case-specific and directly based on data.
Usefulness	Does the explanation provide added value in assisting the trainer’s decision-making?	1 = Not useful at all – provides no guidance for decision-making. 2 = Slightly useful – minimal value, not practically applicable. 3 = Moderately useful – offers some guidance but limited for decision-making. 4 = Useful – supports decision-making with only minor gaps. 5 = Very useful – directly supports decision-making and suggests next steps.

**Model Hyperparameters.** All CatBoost models in this study were trained using consistent hyperparameter settings to ensure comparability across experiments. The number of boosting iterations was set to 100, providing a balance between model complexity and overfitting risk. A learning rate of 0.1 was chosen to enable stable and gradual convergence during training. The maximum tree depth was limited to 6, allowing sufficient model expressiveness while maintaining generalization capabilities. To ensure reproducibility of results, the random seed was fixed at 42 throughout all experiments.

**Validation Strategy.** To obtain robust performance estimates and prevent data leakage, we used an Out-of-Fold (OOF) validation strategy. Specifically, stratified 5-fold cross-validation was employed to ensure consistent class proportions across folds. This approach allowed every instance in the dataset to be used for both training and evaluation, and reduced variance through averaging across five validation runs. Stratification was crucial given the class imbalance (31.7% positive cases), ensuring that each fold was representative of the overall dataset distribution.

**Threshold Optimization.** We adopted a structured approach to threshold optimization aimed at aligning model outputs with both statistical performance goals and practical operational requirements. The process began with *threshold grid generation*, where a dense set of candidate threshold values was created to span the full range between 0 and 1. To enhance search precision, the grid was generated adaptively, with higher point density near the extremes (0 and 1), where predicted probabilities often concentrate in real-world classification settings. This design allowed for a fine-grained examination of model performance in critical decision regions. For each candidate threshold, we carried out *iterative metric computation* by converting the out-of-fold predicted probabilities into binary class labels (0 or 1) and evaluating multiple performance metrics, such as F1-score and Accuracy. To avoid data leakage and to simulate a realistic deployment scenario, we optimized the threshold on a separate validation fold that was not the test set, following the same stratified 5-fold cross-validation procedure. Thus, for each tested fold, another fold was designated as the validation set, ensuring that threshold tuning did not benefit from information contained in the evaluation data. The threshold that maximized the F1-score, balancing Precision and Recall, was designated as the F1-optimal threshold, while a separate search identified the threshold that maximized Accuracy. The resulting optimal thresholds for F1-score were 0.23, 0.25, and 0.26 for the *BCL*, *BCL + LLM (Sentiment Scores)*, and *BCL + LLM (Sentiment Scores and Word Embeddings)* models, respectively. For Accuracy, the optimal thresholds were 0.47, 0.42, and 0.38 for the same models, respectively. This metric-specific optimization allowed us to explore how different decision boundaries influence model performance under varying evaluation priorities.

## Results

We present the overall performance of the three evaluated models in Table 4. AUC and PR-AUC are threshold-independent metrics computed directly from the predicted probabilities. In contrast, the F1-score and Accuracy values reported in the table are computed after applying decision thresholds selected through the threshold optimization procedure.

Given the class imbalance, model performance is primarily interpreted using AUC, PR AUC, and F1-score. The baseline numerical model (BCL) serves as the reference, while the second model enriches the feature set with LLM-derived sentiment scores, and the final model adds word embeddings to these sentiment features. Incorporating sentiment scores produces a consistent improvement across all reported metrics relative to the baseline, indicating that information extracted from trainer comments using LLMs enhances the model’s ability to separate successful and unsuccessful candidates, complementing the structured BCL assessments rather than replacing them. This suggests that most of the predictive value contained in the text is already captured by the sentiment-based features, and that embeddings provide only marginal additional information in this context. Nevertheless, we use the embedding-augmented model for the threshold optimization analysis in order to examine operational trade-offs under a comprehensive feature representation that integrates all available information sources.

**Table 4:** Performance comparison of models using AUC and PR AUC metrics. Bold values indicate the best performance for each metric across the compared models.

**Model**	**AUC**	**PR AUC**	**F1 Score**	**Accuracy**
BCL	0.7303	0.4971	0.5833	0.6845
BCL + LLM (Sentiment Scores)	0.8298	**0.6493**	0.6712	**0.7644**
BCL + LLM (Sentiment Scores and Word Embeddings)	**0.8344**	0.6424	**0.6820**	0.7593

Table 5 presents the results of the threshold optimization procedure for the final model containing both sentiment features and word embeddings. These results allow us to examine how the model performs under different decision-making priorities. In the precision maximization scenario, the model eliminates large number of false positives, ensuring that virtually no unsuitable dogs are admitted to training. However, this comes at the cost of an extremely low recall, meaning that many potentially successful dogs are excluded. In contrast, the fixed-recall scenarios illustrate how the model’s screening efficiency declines as recall requirements increase. At moderate recall levels, a substantial proportion of unsuitable dogs can still be screened out early, but as recall approaches nearly 99%, the model necessarily admits more unsuitable candidates in order to retain all promising ones.

**Table 5:** Performance metrics across different threshold optimization scenarios.

**Metric**	**Optimized Threshold**	**Precision**	**Recall**	**Screened**	**Training Pass**
Precision	0.8096	0.6249	0.1023	77.9314	18.2833
Recall@0.8	0.2981	0.5881	0.7986	74.1144	42.9729
Recall@0.9	0.1836	0.5172	0.9010	60.9532	55.2028
Recall@1	0.0384	0.3361	0.9937	8.8813	93.7322

The *screened* and *training pass* measures provide further insight into the operational implications of these trade-offs. Precision maximization maximizes cost savings by excluding all unsuitable dogs, but drastically reduces the intake of potentially successful candidates, which may limit program capacity. Conversely, fixed-recall scenarios allow program managers to control the balance between risk and efficiency: higher recall thresholds safeguard against missing promising candidates but inevitably increase training costs by admitting more unsuitable ones. Taken together, these results demonstrate that the choice of operating point is not purely a technical consideration but a strategic decision that depends on the organization’s tolerance for risk, its resource constraints, and its broader objectives. The flexibility of the final model across these scenarios suggests it can be adapted to suit a variety of operational policies, but also underscores the importance of aligning threshold selection with specific business priorities.

**Fig. 2:**
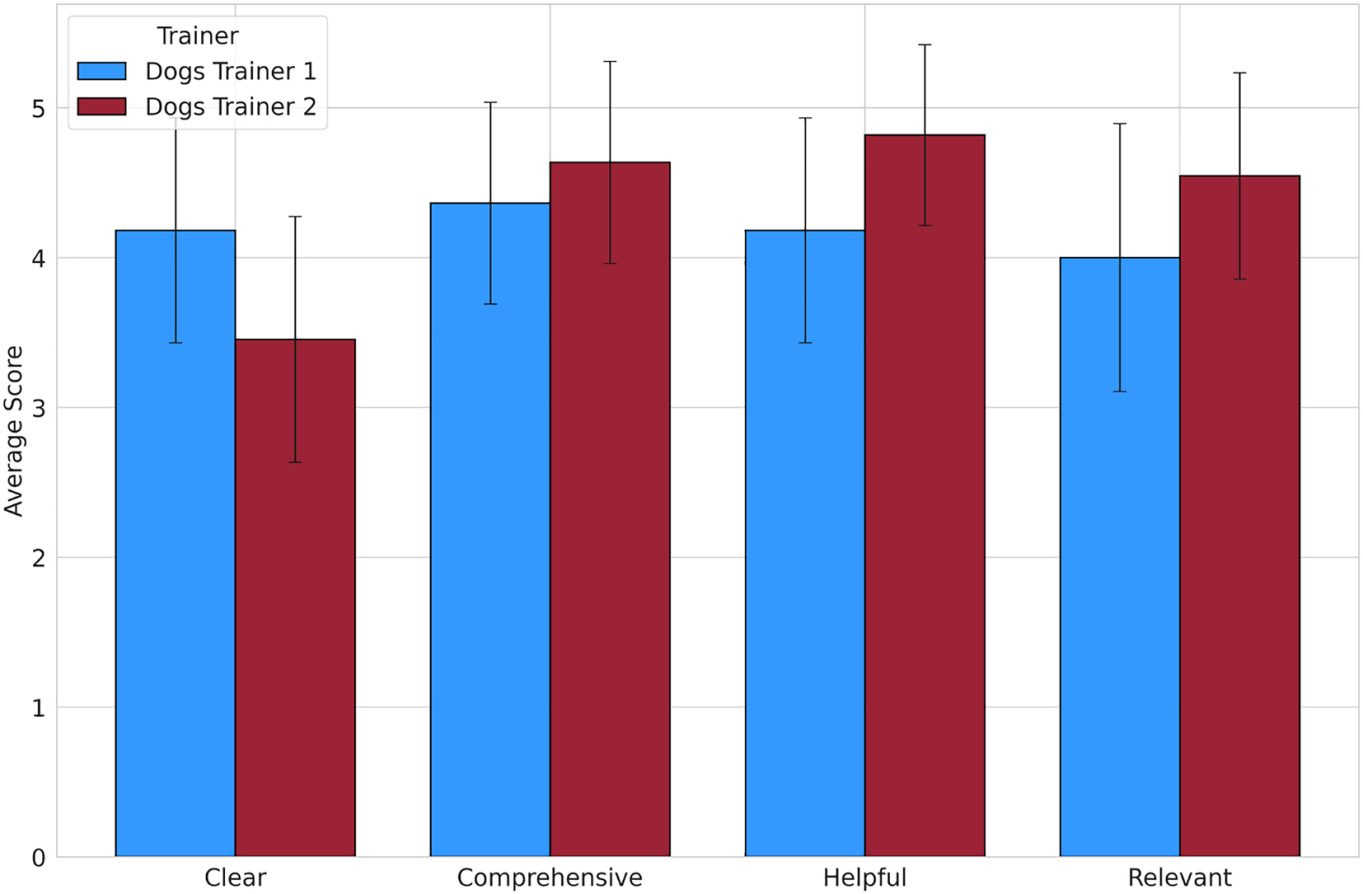
Evaluation results of the explainability questionnaire completed by two experienced dog trainers.

**Explainability Evaluation.** To assess the explainability of our method, we conducted a pilot qualitative assessment based on questionnaire responses completed by two experienced dog trainers. Both trainers were familiar with the 11 dogs included in the classification process and were therefore able to evaluate how realistic and useful the generated explanations were. We present the results of their assessments in Figure 2. We observe that the evaluations indicate that the explanations produced by our method were rated positively across all dimensions. This pilot qualitative assessment is intended to provide initial insights into the perceived relevance, clarity, and usefulness of the generated explanations.

On average, the comprehensiveness score was 4.5, suggesting that the explanations covered most key aspects of the dogs’ suitability for guide work. The clarity score averaged 3.82, indicating that while the explanations were generally understandable, some phrasing could be refined to improve readability and fluency. The relevance score averaged 4.27, showing strong alignment between the explanations and the actual behavioral data of each dog. Finally, the usefulness score was also high, with an average of 4.5, demonstrating that the trainers considered the explanations to be highly supportive in decision-making about each dog’s guide suitability.

We also asked the dog trainers to provide textual feedback on the generated explanations. From their comments, we observed several recurring themes. Trainer 1 noted that some explanations felt “mechanical” or “unnatural” at first but became clearer with familiarity. They emphasized the need for more elegant phrasing, and suggested that the model could highlight key behavioral strengths and weaknesses more explicitly. They also observed occasional mismatches between textual and numerical predictions and proposed displaying graphical summaries to enhance interpretability. Trainer 2 offered constructive suggestions for improving the structure and domain consistency of the reports, recommending that each explanation distinguish between a dog’s strengths and weaknesses, use the terminology of the BCL, and replace numeric scores with qualitative descriptors such as “high” or “low.” They also proposed clustering related behaviors (e.g., stress responses) to create a more cohesive narrative about each dog’s performance.

## Discussion

The first key contribution of this study is the development of a machine learning model for predicting guide dog suitability using in-training BCL scores. To our knowledge, very few prior works have applied predictive AI modelling approaches to forecast outcomes in working dogs, as most research in this domain has relied on traditional statistical analyses rather than modern supervised learning methods.

The most comparable study to ours is that of Amirhosseini et al.^19^, who predicted working dog suitability from structured C-BARQ data collected by puppy raiser families. Their work used two datasets, each more than seven times larger than ours. The first comprised over 7,500 service dogs from diverse working categories, including dogs placed with adults with physical or auditory impairments, children with developmental disabilities, and veterans with post-traumatic stress disorder. The second dataset focused on over 7,500 guide dogs. Their best-performing models achieved 80% accuracy for the service dog dataset and 71% for the guide dog dataset.

In contrast, our model is trained on a much smaller dataset and targets a classification problem most closely aligned with the second dataset of Amirhosseini et al.: distinguishing between guide dogs and non-guide dogs (including both other categories of service dogs and dogs discharged from training). By addressing this narrower but operationally critical task, we demonstrate that meaningful predictive performance can be achieved even with limited data. Specifically, when evaluated using metrics appropriate for imbalanced classification, our best-performing model achieves a AUC of 0.83, a PR-AUC of 0.65, and an F1-score of 0.67, indicating substantial discrimination between successful and unsuccessful candidates and a balanced trade-off between precision and recall in identifying suitable guide dogs.

The second contribution of this study represents the more novel aspect of our work: we provide empirical evidence supporting the hypothesis that free-text notes provided by dog trainers contain behaviorally relevant information that complements, but is not fully captured by, the structured BCL data. This finding is reflected in the improved predictive performance of our model when such free-text information is incorporated alongside the BCL scores.

As part of this contribution, we proposed an LLM-based processing pipeline that converts unstructured trainer notes into numerical feature representations, which can then be fused with structured features such as BCL scores (or, e.g., C-BARQ scores) for suitability prediction. This approach not only leverages recent advances in LLMs for feature extraction but also offers a practical method for fusing heterogeneous data modalities in working dog research. From a more technical perspective, we further investigated the use of abstract semantic embeddings, moving beyond simple sentiment quantification (e.g., counts of positive/negative phrases and their intensity) to capture richer behavioral signals. While these embeddings have the potential to represent higher-level semantics, our experiments showed that they did not consistently add sufficient unique information to justify their added complexity. In certain cases, they even resulted in slightly lower performance compared to the simpler, sentiment-based model, which has the added benefit of being more interpretable to practitioners. We therefore conclude that the proposed embedding method strikes an effective balance between expressiveness and interpretability, capturing the behavioral information needed for suitability prediction without introducing unnecessary computational or conceptual complexity.

Importantly, these performance gains should not be interpreted as evidence that the model uncovers fundamentally new behavioral insights beyond those already recognized by experienced trainers. Rather, the improvement likely reflects the fact that free-text trainer comments contain information that is already implicitly used in day-to-day decision-making, but is not fully represented in structured numerical scores such as the BCL. By transforming these qualitative observations into explicit features, our approach formalizes and translates existing expert knowledge within a machine learning framework. In this sense, the contribution lies in improved information utilization and consistency, rather than in the discovery of new behavioral predictors.

The proposed LLM-based pipeline also includes a component for generating textual explanations aimed at assisting dog trainers in interpreting the model’s predictions. In this study, these explanations were empirically evaluated through structured feedback sessions with two professional dog trainers. While both participants observed that some of the generated explanations exhibited an artificial or overly formal tone characteristic of machine-generated text, they emphasized that the content itself was highly relevant, informative, and aligned with their professional reasoning. This suggests that the explanatory outputs already capture meaningful behavioral insights, even if their phrasing requires refinement. Future work will focus on improving the naturalness, clarity, and contextual sensitivity of these explanations through targeted prompt engineering and fine-tuning of the language model to domain-specific trainer vocabulary and communication styles.

One critical lesson from our work is the importance of selecting evaluation metrics that reflect operational goals. In imbalanced classification problems such as guide dog selection, where the proportion of suitable candidates is relatively small, metrics like AUC-ROC can overstate practical effectiveness because they average performance over all thresholds, many of which are irrelevant in real deployment. Our findings show that threshold-dependent measures such as precision and recall, PR-AUC, offer a clearer picture of how the model will perform under real-world decision rules. Furthermore, incorporating domain-specific indicators such as the percentage of unsuitable dogs screened out and the proportion admitted to training directly links model performance to economic outcomes and resource allocation. This metric-driven perspective ensures that models are not only statistically robust but also operationally valuable, aligning evaluation with the actual costs and benefits experienced by training organizations.

Although our dataset is substantially smaller than those used in comparable studies such as Amirhosseini et al.^19^, our models achieved competitive predictive performance within the context of our task and evaluation setting. In particular, our Accuracy and F1-score values are within the range reported in their study, while our additional use of AUC and PR-AUC provides further evidence of useful ranking and minority-class detection in an imbalanced setting. We emphasize that this comparison is intended as a reference, as the datasets, assessment stage, and labeling schemes differ. This suggests that high-quality, carefully curated data can yield meaningful and generalizable patterns even at a modest scale. Incorporating free-text trainer observations via our LLM-based pipeline appears to partially offset the limitations of dataset size by enriching the feature set with information absent from structured BCL scores. Expanding both the size and diversity of the dataset in future work is likely to further improve predictive accuracy and extend the approach to a wider range of working dog applications.

Building on the findings of this study, future research should focus on expanding the dataset both in size and diversity. Increasing the number of dogs, incorporating datasets from multiple dog training centers, and including a wider range of working dog roles (e.g., service, detection, and therapy dogs) would enhance the generalizability of the models. In parallel, further refinement of the LLM-based text processing pipeline could explore domain-adapted embeddings and hybrid feature extraction approaches to capture even richer behavioral cues from trainer notes. Finally, integrating additional data modalities, such as video-based behavioral assessments, physiological measurements, or wearable sensor data as was done in^14^ may further boost predictive performance and offer deeper insights into the multifaceted nature of working dog suitability.

While the proposed approach is conceptually applicable to other working dog roles and assessment settings, its applicability is likely to depend on the nature of the textual data being integrated. In particular, the value of textual augmentation relies on the expertise of the individuals producing the text, the consistency of their observations, and the existence of a shared evaluative framework. In the present study, trainer comments were authored by professional guide dog trainers operating within a standardized institutional setting, which supports coherence, comparability, and domain relevance of the records. In contexts where textual assessments are provided by non-professional respondents or by highly heterogeneous groups with differing levels of experience or evaluative criteria, the quality and utility of such textual features may be reduced. As such, extending this approach to other domains may require additional considerations, such as calibration of annotators, domain-specific prompt design, or filtering mechanisms to account for variability in writing style and expertise.

## Data Availability

The data used in this study is available from the corresponding author upon reasonable request.

## A Appendix – full prompt for generating explanations

You are an expert in dog behavior and guide dog training. This is about a dog named {name} who is {suit} for the role of a guide dog. The dog’s behavior was evaluated using a predictive model, and the model predicted that the dog would be able to work as a guide dog with a probability of {predict_proba}. As an expert, explain the model’s predictions using the trainer’s notes on the dog’s behavior. Focus on the main issues related to the guide dog: {main_issues_str}. However, briefly and generally note other issues, if any. Use only the handler’s notes on the dog. Do not make up anything extra that is not in the notes. Be brief in your comments. Your answer will be read by the handlers, who are well versed in the topic. Do not go into detail; only describe the dog’s general qualities. For example, if the trainer noted: “Scared by umbrella, moderate recovery. Slight recoil from vacuum cleaner.” Never mention the umbrella, vacuum cleaner, or other specific details! Describe it this way: “Demonstrates mild stress-related avoidance and sensitivity, moderate recovery.” If a problem is not mentioned in the notes, then the dog is doing well. Mention it as a positive quality of the dog and write a coherent text. **IMPORTANT:** Return your response **only** as valid JSON in this exact format: For example, if the trainer notes: “General comments on the evaluation: Excellent test. Very low fear of unfamiliar dogs and moderate fear of unfamiliar people. Resisted collar response. Slight fear of umbrella and narrow passages. Recovered relatively quickly. Minor physical sensitivity to harness. Did not lower his rear end.” Then your JSON response should be: These are the trainer’s notes about {name}: {combined_text}.
